# Assessment of Pansharpening Methods Applied to WorldView-2 Imagery Fusion

**DOI:** 10.3390/s17010089

**Published:** 2017-01-05

**Authors:** Hui Li, Linhai Jing, Yunwei Tang

**Affiliations:** Key Laborary of Digital Earth Sciences, Institute of Remote Sensing and Digital Earth, Chinese Academy of Sciences, Beijing 100094, China; tangyw@radi.ac.cn

**Keywords:** pansharpening, WorldView-2, quality indices, information indices

## Abstract

Since WorldView-2 (WV-2) images are widely used in various fields, there is a high demand for the use of high-quality pansharpened WV-2 images for different application purposes. With respect to the novelty of the WV-2 multispectral (MS) and panchromatic (PAN) bands, the performances of eight state-of-art pan-sharpening methods for WV-2 imagery including six datasets from three WV-2 scenes were assessed in this study using both quality indices and information indices, along with visual inspection. The normalized difference vegetation index, normalized difference water index, and morphological building index, which are widely used in applications related to land cover classification, the extraction of vegetation areas, buildings, and water bodies, were employed in this work to evaluate the performance of different pansharpening methods in terms of information presentation ability. The experimental results show that the Haze- and Ratio-based, adaptive Gram-Schmidt, Generalized Laplacian pyramids (GLP) methods using enhanced spectral distortion minimal model and enhanced context-based decision model methods are good choices for producing fused WV-2 images used for image interpretation and the extraction of urban buildings. The two GLP-based methods are better choices than the other methods, if the fused images will be used for applications related to vegetation and water-bodies.

## 1. Introduction

The WorldView-2 (WV-2) satellite, launched in October 2009, offers eight multispectral (MS) bands of 1.84-m spatial resolution and a panchromatic (PAN) band of 0.46 m spatial resolution [1]. The MS bands cover the spectrum from 400 nm to 1050 nm, and include four conventional visible and near-infrared MS bands: blue (B, 450–510 nm), green (G, 510–580 nm), red (R, 630–690 nm), and near-IR1 (NIR1, 770–895 nm); and four new bands: coastal (C, 400–450 nm), yellow (Y, 585–625 nm), red edge (RE, 705–745 nm), and near-IR2 (NIR2, 860–1040 nm). The PAN band has a spectral response range of 450–800 nm, which covers shorter NIR spectral range than some common PAN bands of 450–900 nm. The WV-2 images have been widely used in various fields, e.g., geological structure interpretation [1], Antarctic land cover mapping [2], bamboo patch mapping [3], high density biomass estimation for wetland vegetation [4], mapping natural vegetation on a coastal site [5], predicting forest structural parameters [6], and especially for the detection of urban objects. Since numerous applications need high-spatial-resolution (HSR) MS images, it is highly desirable to fuse the eight MS bands and the PAN band to produce HSR MS imagery for better monitoring the Earth’s surface. 

Numerous pansharpening methods have been proposed in the last decades to produce spatially enhanced MS images by fusing the MS and PAN images. These methods are divided into two categories: the component substitution (CS) family and multi-resolution analysis (MRA) family. The CS approaches focus on the substitution of a component that is obtained by a spectral transformation of the MS bands with the PAN image. The representative CS methods are the intensity-hue-saturation [7,8], principal component analysis [9], and Gram-Schmidt spectral sharpening (GS) [10,11] methods. The CS methods are easy to implement, and the generated fused MS images yield high spatial quality. However, the CS methods suffer from spectral distortions since the local dissimilarities between the PAN and MS channels, which are caused by different spectral response ranges, are not considered by them. The MRA-based techniques rely on the injection of the spatial details that are obtained through a multi-resolution decomposition of the PAN image into the up-sampled MS bands. Multi-resolution decomposition methods, such as “à trous” wavelet transform [12,13], undecimated or decimated Wavelet transform [14,15,16], Laplacian pyramids [17], Contourlet [18,19,20], and Curvelet [21], are often employed to extract spatial details of the PAN image. Although the MRA-based methods better preserve spectral information of the original MS images than the CS methods, they may cause spatial distortions, such as ringing or aliasing effects, originating shifts or blurred contours and textures [22]. Numerous hybrid schemes combining CS and MRA-based methods are developed to maximize spatial improvement and minimize spectral distortions [23,24,25,26]. In addition, several new pansharpening methods were proposed for the fusion of WV-2 imagery, i.e., the Hyperspherical Color Sharpening (HCS) [27] method and the improved Non-subsampled Contourlet Transform (NSCT) method [28]. These methods were proved to be better than early CS methods, such as GS, PCA.

Several studies have performed comparisons and analyses of some widely used state-of-the-art pansharpening methods, using test images covering different regions from several sensors. Previous studies showed that a pansharpening method may give different performances for test images from different sensors [29,30]. A noticeable point for the WV-2 is that the spectral ranges of the PAN band overlap limited party of the spectral ranges of the C, NIR1, and NIR2 bands. This will result in relative low correlation coefficients between these bands and the PAN bands, which may lead to spectral distortions of the fused version of these bands [31]. Regarding the wide use of the fused WV-2 images, it is urgent to evaluate the performances of different state-of-the-art pansharpening methods applied to WV-2 imagery. Some of the previous comparisons also used test images recorded by WV-2 [30,32,33,34,35] and other sensors. In these works, the early pansharpening methods, such as GS, PANSHARP, Ehlers, modified intensity-hue-saturation (M-IHS), high pass filter (HPF), principal component analysis (PCA), and wavelet-PCA (W-PCA) methods were assessed regarding quality indexes and visual inspection, usually using one or two test images covering urban areas. However, a fusion product providing the best performance in terms of quality indexes and visual inspection may be the best choice for applications such as image interpretation, but it may be not the best choice for applications related to classification and objects identification, i.e., the extraction of buildings, vegetation, and water-bodies [29,36,37]. Consequently, it is important to evaluate the widely used state-of-the-art pansharpening methods from the point of applications, such as land cover classification and object extraction. The purpose of this study was to assess the performances of the existing state-of-the-art pansharpening methods applied to WV-2 imagery, using information indices related to land cover classification and information extraction, as well as quality indexes and visual inspection. Several test images, presenting typical image scenes covering urban, suburban, and rural regions, are employed in the experiments. In addition, the newly proposed HCS, and NSCT methods, which are rarely included in previous comparisons, will be included in this work. 

In this study, eight state-of-the-art algorithms, most of which have been demonstrated to outperform some other methods were assessed using both quality indices and information indices, along with visual inspection. The selected algorithms include four methods belonging to the CS family and four methods belonging to the MRA family. The four CS methods including Gram-Schmidt (GS) [10], adaptive GS (GSA) [38], Haze- and Ratio-based (HR) [39], and HCS [27] were compared. The four MRA methods include undecimated “à trous” wavelet transform (ATWT) using additive injection model [40,41], Generalized Laplacian pyramids (GLP) using spectral distortion minimal model (SDM) and context-based decision model (CBD) [42,43], and the improved NSCT method introduced in [28]. Traditional image quality indices couple with visual inspection were adopted to assess the quality of the fused images. Four comprehensive indices, including Dimensionless Global Relative Error of Synthesis (ERGAS) [44], Spectral Angle Mapper (SAM) [45], *Q*2^n^ [46,47], and spatial correlation coefficient (SCC) [48] were employed to measure the spectral distortion between the fused and the original MS bands. Regarding the application purpose of the high-resolution fused images, which includes land cover classification of urban or suburban areas, bamboo and forest mapping, and so on, some widely used indexes, derived from the fusion products, were assessed to evaluate the information presentation ability of the fusion products. The employed indexes include morphological building index (MBI) [49], normalized difference vegetation index (NDVI), and normalized difference water index (NDWI). The information presentation of a fusion product was assessed using the correlation coefficient (CC) between an index derived from the fusion product and the same index derived from the corresponding original MS image. A higher CC value implies a better information preservation ability of the fusion product.

This paper is organized as follows: the eight selected pansharpening methods are introduced in Section 2, as well as the quality indexes; the experimental results with visual and quantitative comparisons with other outstanding fusion methods are presented in Section 3. Discussions are presented in Section 4, whereas the conclusions are summarized in Section 5.

## 2. Methodology

### 2.1. Algorithms

The algorithms used for the comparisons are introduced in the following subsections. *MS* and *P* represent the original low-resolution MS image and high-resolution PAN image, respectively. $\tilde{MS}$ and $\hat{MS}$ represent the up-sampled MS and the fused MS images, respectively. A general formulation of CS fusion is given by:

(1) $${\hat{MS}}_{i}={\tilde{MS}}_{i}+g_{i}\left(P-I_{L}\right),i=1,\ldots ,N$$

in which the subscript *i* indicates the *i*th spectral band; $g_{i}$ is the injection gains of the *i*th band, while the intensity image $I_{L}$ is defined by Equation (2):

(2) $$I_{L}=\;\overset{N}{\underset{i=1}{\sum }}w_{i}\tilde{{\mathrm{MS}}_{i}}$$

where *w*_i_ is the weight of the *i*th MS band, and *N* is the number of MS bands. 

Similarity, a general formulation for MRA-based methods can be given by Equation (3):

(3) $${\hat{MS}}_{i}={\tilde{MS}}_{i}+g_{i}\left(P-P_{L}\right),i=1,\ldots ,N$$

where *P*_L_ is the low-frequency component of the PAN band. *P*_L_ can be obtained by different approaches, such as low-pass filter, Laplacian pyramid and wavelet decomposition.

#### 2.1.1. GS and GSA

GS is a representative method of the CS family, the fusion process of which is described by Equation (1), with the injection gains $g_{i}$ given by Equation (4):

(4) $$g_{i}=\;\frac{\mathrm{cov}\left(MS_{i},I_{L}\right)}{\mathrm{var}\left(I_{L}\right)}$$

where cov (***X***, ***Y***) is the covariance between the two images ***X*** and ***Y***, and var (***X***) is the variance of ***X***. 

Several versions of GS can be achieved by changing the method for generating *I*_L_. One way to obtain *I*_L_ is simply averaging the MS components (i.e., using Equation (2) with setting $w_{i}=1$ for all i=1,…,N). This modality is referred as GS or GS mode 1. Another way is using the low-pass version of *P* as *I*_L_. This modality is referred as GS mode 2.

An enhanced version, called adaptive GS (GSA), is proposed by assigning *I*_L_ as a weighted average of the original MS bands, as Equation (2). The weights *w_i_* in Equation (2) are calculated with the minimum mean square error (MSE) solution of Equation (5):

(5) $$P_{0}=\;\overset{N}{\underset{i=1}{\sum }}w_{i}MS_{i}+\varepsilon$$

where $P_{0}$ is the degraded version of *P*, with the same pixel sizes of the original MS bands. $P_{0}$ is generated by low-pass filtering of *P*, followed by decimation. Both the GS and GSA methods are included in the experiments in this study.

#### 2.1.2. HR

The HR method is based on the assumption that the ratio of a HSR MS band to a low-spatial-resolution (LSR) MS band is equal to the ratio of a HSR PAN image to an assumed LSR PAN image a fusion method that considers haze [50,51,52]. The fused *i*th MS band ${\hat{MS}}_{i}$ can be calculated by using Equation (6) according to HR fusion method:

(6) $${\hat{MS}}_{i}=\left({\tilde{MS}}_{i}-H_{i}\right)\frac{P-H_{p}}{P_{L}-H_{p}}+H_{i},i=1,\ldots ,N$$

where $P_{L}$ is a low-pass version of *P*; *H_i_* and *H*_p_ denote the haze values in the *i*th MS band and the PAN band, respectively. The values of *H_i_* and *H*_p_ can be determined using the minimum grey level values in $MS_{i}$ and *P* according to an image-based dark-object subtraction method [50,51,52].

#### 2.1.3. HCS

HCS is a pansharpening method designed for WV-2 imagery based on transforming the MS bands into hyperspherical color space. The HCS method offers two modes, including the native mode and the smart mode. The process of the native mode is described as follows:

(a) The squares of the multispectral intensity (*I*^2^) and the PAN (*P*^2^) are calculated using Equations (7) and (8), respectively:
  (7) $$I^{2}=\overset{N}{\underset{i=1}{\sum }}{{\tilde{MS}}_{i}}^{2}$$
  (8) $$P^{2}={\left(P\right)}^{2}$$
(b) Calculate the mean (*u_P_*) and standard deviation (*σ_p_*) of *P*^2^, as well as the mean (*u*_I_) and standard deviation (*σ*_P_) of *I*^2^.
(c) The *P*^2^ is adjusted to the mean and standard deviation of *I*^2^, using Equation (9):
  (9) $$P^{2}=\;\frac{\sigma_{I}}{\sigma_{P}}\left(P^{2}-u_{P}+\sigma_{P}\right)+u_{I}-\sigma_{I}$$
(d) The square root of the adjusted *P*^2^ is assigned to *I*_adj_ (i.e., $I_{adj}=\sqrt{P^{2}}$), *I*_adj_ is used in the reverse transform from HCS color space back to the original color space, using Equation (10):
  (10) $${\tilde{MS}}_{i}=\{\begin{array}{cc} I_{adj}cos\varphi_{i}, & i=1\\ I_{adj}sin\varphi_{1}sin\varphi_{2}\ldots sin\varphi_{i-1}cos\varphi_{i}, & i=2,\ldots ,N-1\\ I_{adj}sin\varphi_{1}sin\varphi_{2}\ldots sin\varphi_{i-1}, & i=N \end{array},$$
  in which $\varphi_{i}$ is defined using Equation (11):
  (11) φi={tan−1(∑k=2NMS˜k2MS˜i),i=1tan−1(∑k=i+1NMS˜k2MS˜i),i=2,…,N−2tan−1(MS˜i+1MS˜i),i=N−1

For the smart case, similarity to *P*^2^, a *PS*^2^ is calculated by using the low-pass version of the PAN image (*P*_L_), i.e., $PS^{2}={\left(P_{L}\right)}^{2}$. The *P*_L_ is generated by an average filtering with size of 7 × 7. Both the means and standard deviations of *PS*^2^ and *P*^2^ are adjusted to those of *I*^2^. Then, the adjusted intensity *I*_adj_ is assigned by using Equation (12):

(12) Iadj=(P2PS2)I2

Finally, the fused image can be generated by using Equations (10) and (11). The HCS method using the smart mode is considered in the experiment in this study.

#### 2.1.4. ATWT

The ATWT method is a MRA fusion approach that extracting spatial details using “à trous” wavelet transform. The fusion scheme utilizing the additive injection model can be formulated as:

(13) $${\hat{MS}}_{i}={\tilde{MS}}_{i}+P-P_{L},i=1,\ldots ,N$$

where *P*_L_ is the low frequency component of *P* and is generated by the “à trous” wavelet. 

The “à trous” wavelet is a kind of non-orthogonal wavelet that is different from orthogonal and biorthogonal. It is a redundant transform, since decimation is not implemented during the process of wavelet transform while the orthogonal or biorthogonal wavelet can be carried out using either decimation or undecimation mode. 

The whole process of the ATWT fusion can be divided into two steps [40]:

(1) Use the à trous wavelet transform to decompose the PAN image to *n* wavelet planes. Usually, *n* = 2 or 3.
(2) Add the wavelet planes (i.e., spatial details) of the decomposed PAN images to each of the spectral bands of the MS image to produce fused MS bands.

#### 2.1.5. GLP

The fusion process of GLP can also be formulated as Equation (3). For the GLP method, the low-frequency component of the PAN band *P*_L_ is generated by up-sampling the down-sampled version of the original PAN band *P*. The down-sampling of *P* is implemented using a low-pass reduction filter that matches the MTF of the band, whereas the up-sampling of *P* is carried out by using an ideal expansion low-pass filter.

For the GLP fusion, different detail injection models are designed to obtain the injection coefficients *g_i_*. The most used models are the spectral distortion minimizing (SDM) model and the context-based decision (CBD) model. 

For the SDM model, the injection coefficients *g_i_* can be obtained using Equation (14):

(14) $$g_{i}\left(m,n\right)=\beta (m,n)\frac{{\tilde{MS}}_{i}(m,n)}{P_{L}(m,n)},i=1,\ldots ,N$$

where β(m,n) is equal to 1 for all pixels in the original SDM model [42], whereas β(m,n) is defined as the ratio between average local standard deviations of resampled MS bands and local standard deviation of *P*_L_ , for the enhanced SDM (ESDM) model proposed by Aiazzi [43]:

(15) $$\beta (m,n)\sqrt{\frac{\frac{1}{N}\sum_{k=1}^{N}var[{\tilde{MS}}_{k}](m,n)}{var[P_{L}](m,n)}}$$

In the CBD model, the space-varying coefficients *g_i_* is defined as Equation (16):

(16) $$g_{i}\left(m,n\right)=\{\begin{array}{cc} \min\left\{\frac{\sigma_{{\tilde{MS}}_{i}}\left(m,n\right)}{\sigma_{P_{L}}\left(m,n\right)},c\right\} & if\;\rho_{i}\left(m,n\right)\geq \theta_{i},\\ 0 & if\;\rho_{i}\left(m,n\right)<\theta_{i}, \end{array}$$

in which $\rho_{i}\left(m,n\right)$ is the local correlation coefficient between ${\tilde{MS}}_{i}$ and *P*_L_ calculated on a square sliding window of size *L* × *L* centered on pixel (*m*, *n*). 

The CBD model is uniquely defined by the set of thresholds $0<\theta_{i}\leq 1$, for i=1,…,N, generally different for each band, and by the window size *L* depending on the spatial resolutions and scale ratio of the images be merged, as well as the landscape characteristics (typically, 7≤L≤11 to avoid loss of local sensitivity with *L* > 11 and statistical instability with *L* < 7). The thresholds may be related to the spectral content of the Pan image, e.g., $\theta_{i}=1-\rho_{i}$, where $\rho_{i}$ is the global correlation coefficient between the *k*th band and the Pan image spatially degraded to the same resolution. A clipping constant *c* was introduced to avoid numerical instabilities (empirically, 2≤c≤3). 

For the Enhanced CBD (ECBD) model proposed by Aiazzi [43], the coefficients $g_{i}$ is calculated as Equation (17):

(17) $$g_{i}\left(m,n\right)=\min\left\{\frac{\sigma_{{\tilde{MS}}_{i}}\left(m,n\right)}{\sigma_{P_{L}}\left(m,n\right)}\cdot \frac{\rho_{i}\left(m,n\right)}{\bar{\rho_{i}}},c\right\}$$

where $\bar{\rho_{i}}$ is the global correlation coefficient between ${\tilde{MS}}_{i}$ and $P_{L}$. Both the GLP methods using the ESDM and the ECBD models are considered in the experiment.

#### 2.1.6. NSCT

The contourlet transform (CT) is proved to be a better approach than the wavelet for pan-sharpening. CT is implemented by a multiscale decomposition using the Laplacian pyramid followed by a local directional transform using the directional filter bank (DFB). 

The NSCT is a shift-invariant version of CT and has excellent multilevel and multi-direction properties. NSCT is built upon the non-subsampled pyramid filter banks (NSPFBs) and the non-subsampled directional filter banks (NSDFBs). The NSPFB employed by NSCT is a 2-D two-channel non-subsampled filter bank, whereas the NSDFB employed by NSCT is a shift-invariant version of the critically sampled DFB in CT. The details of the NSCT can be attended in [28,53]. An improved version of the standard NSCT-based method is introduced in [28]. The process of the improved NSCT method with mode 2 (NSCT_M2) in [28] is descripted as follows.

(a) Each original MS band $MS_{i},$ is decomposed using 1-level NSCT to get one coarse level, $C_{0}^{MS_{i}}$, and one fine level, $C_{1}^{MS_{i}}$;
(b) The PAN band is decomposed using3-level NSCT into one coarse level, $C_{0}^{PAN}$, and three fine levels, which are denoted as $C_{1}^{PAN}$, $C_{2}^{PAN}$, and $C_{3}^{PAN}$, respectively.
(c) The coefficients of each MS band, $C_{0}^{MS}$ and $C_{1}^{MS}$, are up-sampled to the scale of the PAN band using the bi-linear interpolation algorithm.
(d) The coarse level of the fused *i*th MS band, $C_{0}^{F_{i}}$, is the up-sampled coarse level of the *i*th MS band $C_{0}^{MS_{i}}$, whereas the fine levels 2 ($C_{2}^{F_{i}}$) and 3 ($C_{3}^{F_{i}}$) of the fused *i*th MS band are the fine levels 2 ($C_{2}^{PAN}$) and 3 ($C_{3}^{PAN}$) of the PAN band.
(e) The fused fine level 1, $C_{1}^{F_{i}}$, is obtain by fusing the coefficients of the same level obtained from both the *i*th MS band and the PAN band. For each pixel (*x*, *y*), the coefficients of the fused fine level 1, $C_{1}^{F_{i}}\left(x,y\right)$, is determined according to Equation (18):
  (18) $$C_{1}^{F_{i}}\left(x,y\right)=\{\begin{array}{cc} C_{1}^{MS_{i}}\left(x,y\right), & LE_{MS_{i}}\left(x,y\right)>LE_{\mathrm{PAN}}\left(x,y\right)\\ C_{1}^{PAN}\left(x,y\right), & otherwise \end{array}$$
  where $LE_{MS_{i}}\left(x,y\right)$ and *LE*_PAN_(*x*, *y*) are the local energy of pixel (*x*, *y*) for the *i*th MS band and the PAN band, respectively, calculated within a (2*M* + 1) × (2*P* + 1) window using the formula shown in Equation (19):
  (19) $$LE\left(x,y\right)=\overset{M}{\underset{i=-M}{\sum }}\overset{P}{\underset{j=-P}{\sum }}{\left(C_{1}\left(x+i,y+j\right)\right)}^{2}$$
  The inverse NSCT is applied to the fused coefficients to provide the fused *i*th MS band.
  This improved version was demonstrated to provide pansharpened images with a good spectral quality.

### 2.2. Quality Indexes

Quality assessment of fusion products can be performed using two approaches. The first approach considers fusing images at a spatial resolution lower than the original resolution and uses the original MS image as a reference to assess the quality of the fused images. Several indexes have been proposed for evaluating the spatial and spectral distortions of the fused image with respect to an available reference image. The widely used spectral quality indexes include Root Mean Square Error (RMSE), Relative Average Spectral Error (RASE) [54], ERGAS, SAM, Universal Image Quality Index (UIQI) [55], Q4 [46], Q2n, and Peak Signal-to-Noise Ratio (PSNR) [56], whereas the widely used spatial indexes include SCC and Structural SIMilarity (SSIM) [57]. The second approach uses quality indexes that do not require a reference image but operate on relationships among the original images and the fusion products. This approach has the advantage of validating the products at the original scale, thus avoiding any hypothesis on the behavior at different scales. However, appropriate indexes requiring no reference should be exploited to assess the quality of the fusion product. The Quality with no reference index (QNR) was one of the mostly used indexes [58]. It is composed by the product of two separate values that quantify spectral and spatial distortions, respectively. However, QNR is proved to be lower reliability than the indexes belonging to the first approach [30], since it can be affected by slightly mismatches among the original image bands. The acquisition modality of WV-2 can led to s mall temporal misalignments among the MS bands, since the eight MS bands of are arranged in two arrays of four bands each. Consequently, the QNR index is not used in this study. Four quality indexes belong to the first approach, including ERGAS, SAM, Q2n, and SCC, are employed to assess the quality of fused images at data level. These indexes are chosen due to they are widely used in literatures related to fusion of remote sensing imagery.

#### 2.2.1. ERGAS

The global index ERGAS is an improved version of the index named Relative Average Spectral Error (RASE), which is defined based on Root Mean Square Error (RMSE). The formula of ERGAS [44] is defined as follows:

(20) $$\mathrm{ERGAS}=\frac{100}{R}\sqrt{\frac{1}{N}\overset{N}{\underset{k=1}{\sum }}{\left(\frac{RMSE\left({\hat{MS}}_{k},MS_{k}\right)}{u\left(MS_{k}\right)}\right)}^{2}}$$

where $u\left(MS_{k}\right)$ is the mean of the *k*th band of the reference image; *R* is the spatial resolution ratio between the MS and PAN bands; RMSE is defined as Equation (21):

(21) $$RMSE\left({\hat{MS}}_{k},MS_{k}\right)=\sqrt{E\left[{\left({\hat{MS}}_{k}-MS_{k}\right)}^{2}\right]}$$

The optimal value for ERGAS is 0, since it is defined as a weighted sum of RMSE values.

#### 2.2.2. SAM

SAM expresses the spectral similarity between a fused image and a reference image with the average spectral angle of all pixels involved [45]. Let two spectral vectors $V=\left\{V_{1},V_{2},\ldots ,V_{N}\right\}$ and $\hat{V}=\left\{{\hat{V}}_{1},{\hat{V}}_{2},\;\ldots ,{\hat{V}}_{N}\right\}$ present the reference spectral pixel and the fused spectral pixel, respectively, their spectral angle SAM is defined as in Equation (22):

(22) $$\mathrm{SAM}=\arccos\left(\frac{\langle V,\hat{V}\rangle }{\left|V\right|\cdot \left|\hat{V}\right|}\right)$$

where 〈X,Y〉 stands for the inner-product of the two vectors X and Y, and |X| stands for the modulus of a vector X. 

The smaller the spectral angle, the higher the similarity between the two vectors. Since the angle is independent of the magnitudes of the two vectors, the index SAM is not affected by solar illumination factors.

#### 2.2.3. Q2^n^

Q2^n^ is a generalization of Q index for monoband images [47] and an extension of Q4 [46]. Q2^n^ is derived from the theory of hypercomplex numbers, particularly of 2^n^-ones [59,60]. For a 2^n^-on hypercomplex random variable *z* (in boldface) is written in the following form:

(23) $$z=z_{0}+i_{1}\cdot z_{1}+\ldots +i_{2^{n}-1}\cdot z_{2^{n}-1}$$

where $z_{0},z_{1},\ldots ,z_{2^{n}-1}$ are real numbers, and $i_{0},i_{1},\ldots ,i_{2^{n}-1}$ are hypercomplex unit vectors, the conjugate $z^{*}$ is given by:

(24) $$z^{*}=z_{0}-i_{1}\cdot z_{1}-\ldots -i_{2^{n}-1}\cdot z_{2^{n}-1}$$

and the modulus |z| is defined by Equation (25):

(25) $$\left|z\right|=\sqrt{z_{0}^{2}+z_{1}^{2}+\ldots +z_{2^{n}-1}^{2}}$$

Give two 2^n^-on hypercomplex random variables ***z*** and ***v***, the hypercomplex covariance between ***z*** and ***v*** is defined as Equation (26):

(26) $$cov\left(z,v\right)≜E\left[\left(z-\bar{z}\right)\left(v-\bar{v}\right)\right]=E\left[zv^{*}\right]-\bar{z}\cdot {\bar{v}}^{*}=\sigma_{z,v}$$

The hypercomplex CC between the two 2^n^-on random variables are defined as the normalized covariance:

(27) $$q\left(z,v\right)≜\frac{\sigma_{z,v}}{\sigma_{z}\sigma_{v}}$$

in which $\sigma_{z}$ and $\sigma_{v}$ are the square roots of the variances of ***z*** and ***v***, and are obtained by Equations (28) and (29), respectively:

(28) $$\sigma_{z}=E\left[{\left|z\right|}^{2}\right]-{\left|\bar{z}\right|}^{2}$$

(29) $$\sigma_{v}=E\left[{\left|v\right|}^{2}\right]-{\left|\bar{v}\right|}^{2}$$

The index Q2^n^ can be computed from Equation (30):

(30) $$Q{2^{n}}_{M\times M}=\frac{\sigma_{z,v}}{\sigma_{z}\sigma_{v}}\cdot \frac{2\bar{z}\bar{v}}{{\left(\bar{z}\right)}^{2}+{\left(\bar{v}\right)}^{2}}\cdot \frac{2\sigma_{z}\sigma_{v}}{{\sigma_{z}}^{2}+{\sigma_{v}}^{2}}$$

where *M* is the size of the local window used to calculate $Q2^{n}$. 

Finally, Q2^n^ is obtained by averaging the magnitudes of all $Q{2^{n}}_{M\times M}$ over the whole image, according to Equation (31):

(31) $$Q2^{n}=E\left[\left|Q{2^{n}}_{M\times M}\right|\right]$$

According to [46], the value of *M* is suggested to be 32.

#### 2.2.4. SCC

To assess the spatial quality of the fusion products, the spatial details presented in the fused images will be compared with those presented in the reference image by calculating the correlation coefficient between the spatial details extracted from the two images. Similar to the procedure proposed by Otazu et al. [48], the spatial information presented in the two images to be compared is extracted by using a Laplacian filter, then, the correlations between the two filtered images are calculated band by band. However, an overall correlation coefficient of the two edge images with eight bands is calculated in this study. A high SCC value indicates that many of the spatial details of the reference image are presented in the fused image.

### 2.3. Information Indexes

In order to assess the ability of information extraction of the fusion products employed in specific remote sensing applications, we assessed the quality of fused images by the use of a series of information indices, which are proved to be useful in land cover classification and information extraction in previous studies. Three indices are employed in this study: MBI, NDVI, and NDWI. The accuracy of an information index derived from a fusion product is assessed using the CC between the information index and the same information index derived from the corresponding reference MS image. A higher CC value implies a better information preservation ability of the fusion product in terms of the information index. Henceforth, the CC values calculated for MBI, NDVI, and NDWI are denoted as *C*_MBI_, *C*_NDVI_, and *C*_NDWI_, respectively. The employed three information indices are introduced in this subsection.

#### 2.3.1. NDVI

Based on the principle that vegetation has a strong reflectance in the near-infrared (*NIR*) channel but a strong absorption in the red (*Red*) channel, the NDVI is defined as Equation (32):

(32) $$NDVI=\frac{NIR-Red}{NIR+Red}$$

#### 2.3.2. NDWI

The NDWI is defined using the spectral value of the Green band (G) and the NIR band as Equation (33):

(33) $$NDWI=\frac{G-NIR}{G+NIR}$$

#### 2.3.3. MBI

The MBI is proposed by Huang et al. [49], aiming to represent spectral and spatial characteristics of buildings using a series of morphological operators. The MBI is calculated according to the following steps:

(a) A brightness image *b* is generated by setting the value of each pixel *p* to be the maximum digital number of the visible bands. Only the visible channels are considered due to they have the most significant contributions to the spectral property of buildings.
(b) The directional white top-hat (WTH) reconstruction is employed to highlight bright structures that have a size equal to or smaller than the size of the structure element (SE), and meanwhile suppresses other dark structures in the image. WTH with linear SE is defined as Equation (34):
  (34) $$WTH\left(s,\theta \right)=b-\gamma_{SE}\left(s,\theta \right)$$
  in which $\gamma_{SE}\left(s,\theta \right)$ is an opening by reconstruction operator using a linear SE with a size of *s* and a direction of θ.
(c) The difference morphological profiles (DMP) of white top-hat transforms are employed to model building structures in a multi-scale manner:
  (35) $$\{\begin{array}{c} DMP_{WTH}=\{DMP_{WTH}\left(s,\theta \right):s_{min}\leq s\leq s_{max},\theta \in D\}\\ DMP_{WTH}\left(s,\theta \right)=\left|WTH\left(s+\Delta s,\theta \right)-WTH\left(s,\theta \right)\right| \end{array}$$
(d) Finally, MBI is defined based on DMP using Equation (36):
  (36) $$MBI=\frac{\sum_{s,dir}DMP_{WTH}\left(s,\theta \right)}{N_{D}\times N_{s}}$$
  where *N*_D_ and *N*_S_ are the directionality and the scale of the DMPs, respectively. The definition of the MBI is based on the fact that building structures have high local contrast and, hence, have larger feature values in most of the directions of the profiles. Accordingly, buildings will have large MBI values.

## 3. Experimental Results

### 3.1. Datasets

**Figure 1:**
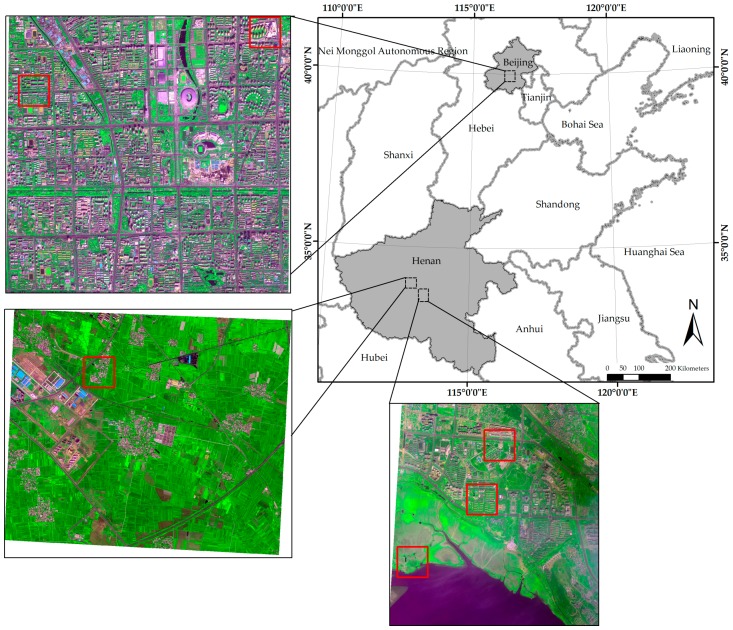
The locations of the three WV-2 scenes employed in this work. The locations of the six test images used in the experiments are marked by the rectangles in red.

**Figure 2:**
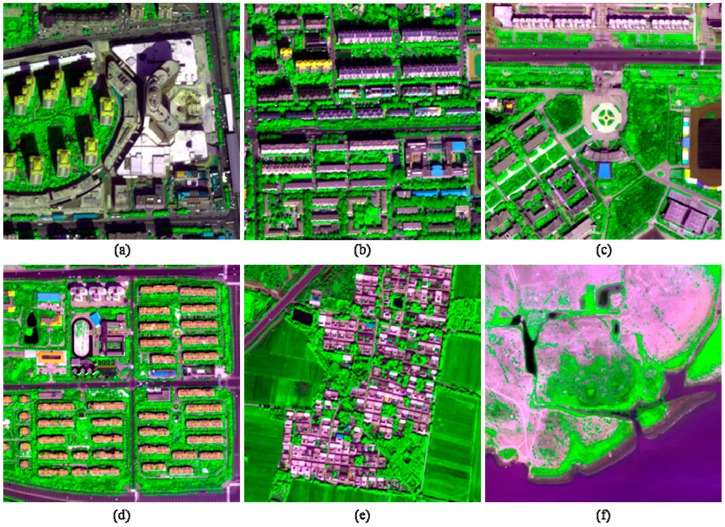
The selected six dataset used in the experiments. (**a**) I1; (**b**) I2; (**c**) I3; (**d**) I4; (**e**) I5; (**f**) I6.

Six datasets clipped from three WV-2 scenes were considered in this study. One scene covers Beijing City, China, whereas the other two scenes cover Pingdingshan City, Hebei Province, China. The Bejing scene was acquired on 21 September 2013, with an off-nadir angle of 13.7°. One of the Hebei scenes was acquired on 29 April 2014, with an average off-nadir angle of 12.5°, whereas the other was obtained on 21 August 2014, with an average off-nadir angle of 8.6°. The locations of the three scenes are shown in Figure 1. The red rectangles in the figure indicate the locations of the six datasets. Two datasets covering urban areas were selected from the Beijing scene, whereas the other four datasets were obtained from the other two WV-2 scenes. Two of them cover suburb areas, whereas the other two cover rural areas. For all the datasets, the radiometric resolution is 16 bits, whereas the spatial resolution ratio *R* is 4. Each dataset has a size of 256 × 256 pixels at MS scale. The two urban images are henceforth referred as I1 and I2, respectively, whereas the two suburban images are referred as I3 and I4, respectively. The two rural images are referred as I5 and I6, respectively. The typical image objects shown in the six images are listed in Table 1; the six images are shown in Figure 2. 

To evaluate the quality of the fusion products with respect to the original MS images, the degraded datasets were produced by reducing the original MS and PAN images to a spatial resolution of 8 m and 2 m, respectively. All the fusion experiments were performed on the degraded datasets. The fused 2-m images were compared with the 2-m true MS images to assess their quality. The degraded images were generated using averaging in this study [61]. The spatial resolution ratio *R* in this study is 4 for all the tested images.

According to the image objects included in the six test images, different information indices were chosen for each of the images (Table 1). The fusion products of the two urban images were assessed with respect to C_MBI_ and C_NDVI_, due to no water bodies can be observed from these two images. The fusion products of the two suburban images were assessed using all the three indices, whereas the fusion products of the two rural images were assessed using only *C*_NDVI_ and *C*_NDWI_.

### 3.2. Fusing Using the Selected Algorithms

All the fusion algorithms were implemented in MATLAB version 2014b. For all the selected eight fusion methods, the up-sampled MS images were produced using the bi-cubical interpolation approach. For the HR method, the low-spatial-resolution version of PAN image (*P*_L_) was obtained by averaging the PAN pixels in an *R* × *R* window, followed by the bi-cubically up-sampled to the resolution of the original PAN image. The GLP methods using the ESDM model and the ECBD model were used in the fusion experiment; the two methods are referred as GLP_ESDM and GLP_ECBD, respectively, in this study. For the HR method, the haze values for the MS and PAN bands were determined using the values of the pixel that offering the lowest value in the PAN image. 

### 3.3. Quality Indexes

#### 3.3.1. Assessment for the Two Urban Images

**Figure 3:**
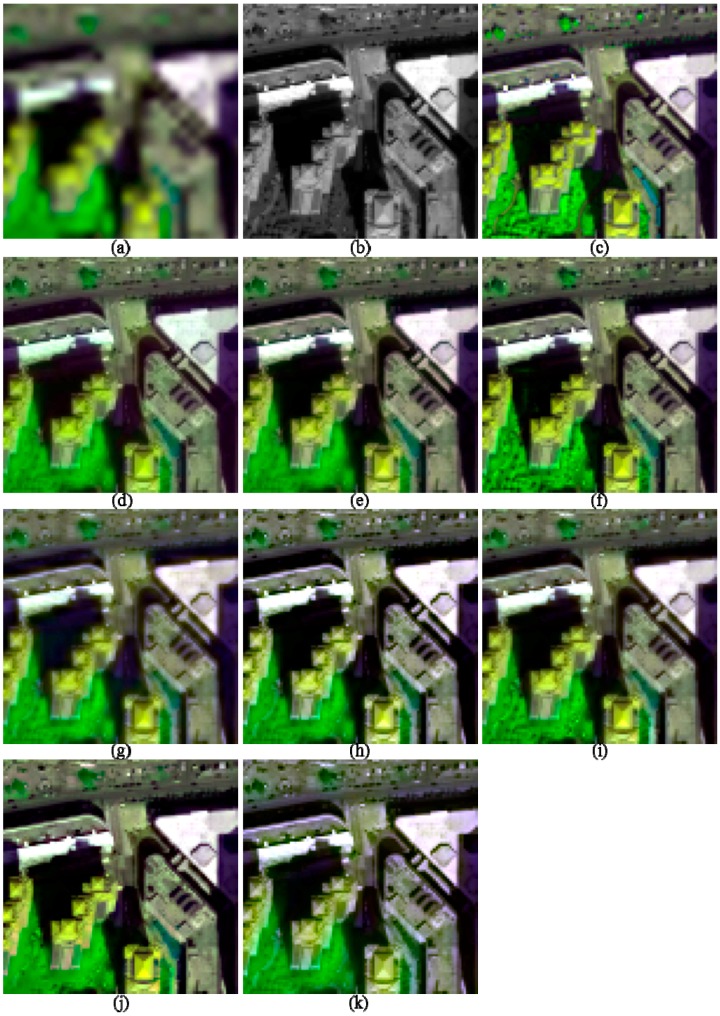
The degraded and 2-m fused images of I1, shown in band 5-7-2 composition. (**a**) 8-m MS; (**b**) 2-m PAN; (**c**) True 2-m MS; (**d**) GS; (**e**) GSA; (**f**) HR; (**g**) HCS; (**h**) ATWT; (**i**) GLP_ESDM; (**j**) GLP_ECBD; and (**k**) NSCT_M2.

**Figure 4:**
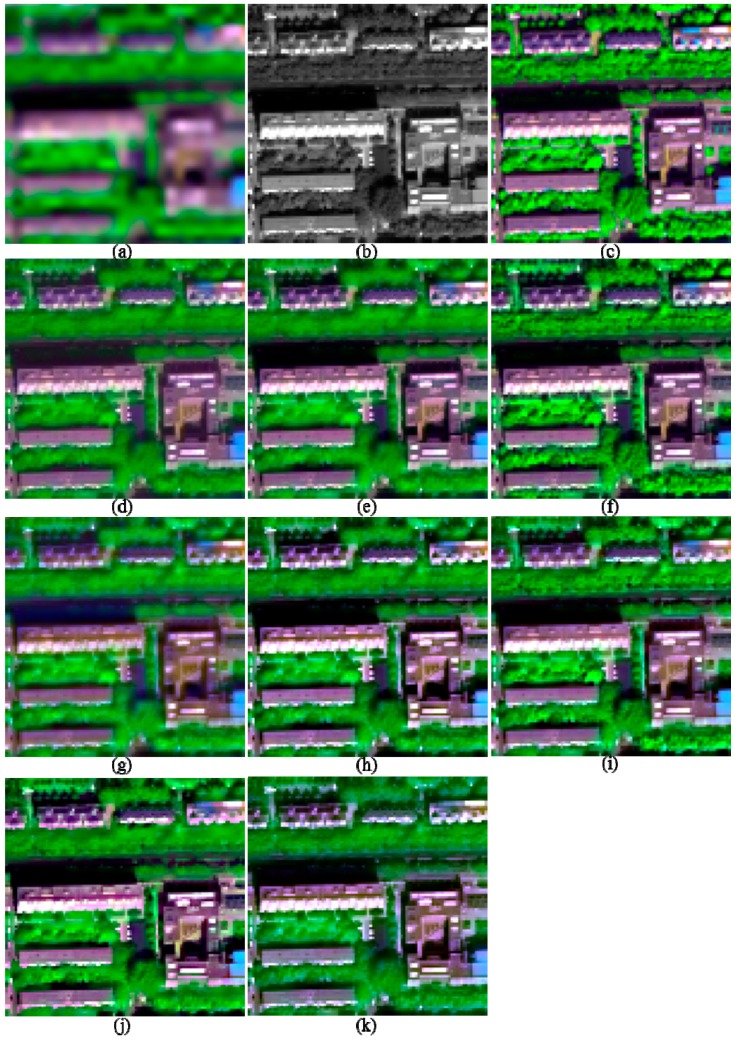
The degraded and 2-m fused images of I2, shown in band 5-7-2 composition. (**a**) 8-m MS; (**b**) 2-m PAN; (**c**) True 2-m MS; (**d**) GS; (**e**) GSA; (**f**) HR; (**g**) HCS; (**h**) ATWT; (**i**) GLP_ESDM; (**j**) GLP_ECBD; and (**k**) NSCT_M2.

The image quality indices of the fusion products of the two urban images are shown in Table 2. The fusion products of these two images are partly shown in Figure 3 and Figure 4, respectively, in order to facilitate the observation of the details of the fused images. The images in each figure in this work are stretched by using an identical histogram obtained from the corresponding reference MS images. 

The HR method offers the highest Q8 and SCC values and the lowest ERGAS and SAM values for the two urban images, indicating that the HR method gives the best performances in both spectral and spatial quality indices. The NSCT_M2 methods yields the lowest Q8 and SCC values, the highest ERGAS and SAM values, indicating the poorest performance in spectral preservation. The GSA method provides Q8 values slight lower than the highest values provided by the HR method, followed by the GS, GLP_ESDM, GLP_ECBD, ATWT, and HCS methods. The GLP_ESDM performs the best among the MRA methods in terms of both spectral and spatial quality indices. The HCS method yields the poorest performance among the CS methods, in terms of all the four quality indices for the two urban images.

Visual comparisons for the fusion products of the two urban images (Figure 3 and Figure 4) show that the fusion products generated by the GS, HCS, and NSCT_M2 methods show significant spectral distortions in shadow covered regions; this is especially obvious for the fusion products of I2. In addition, the fused image generated by the GLP_ECBD method for I2 also show significant spectral distortions in shadow covered regions, which is consistent with the poor performance of the GLP_ECBD method in terms of quality indexes. Although the fused images generated by the ATWT and GLP_ECBD methods show more sharpened boundaries between different objects than the other fusion products, they seem to be over sharpened and show spectral distortions. This is very obvious for the fusion products of I2. The two HCS-fused images are more blurred than other fusion products. It also can be observed that the fusion products generated by GSA, HR, and GLP_ESDM methods yield lower spectral distortions and more sharpened boundaries between different objects than other fusion products. In addition, the HR-fused images provide more texture details in vegetation covered areas.

According to the quality indexes and visual inspection, the HR, GSA, and GLP_ESDM methods give better performances than the other methods, whereas the NSCT_M2 and HCS methods offer the poorest performances, for the two urban images.

#### 3.3.2. Assessment for the Two Suburban Images

**Figure 5:**
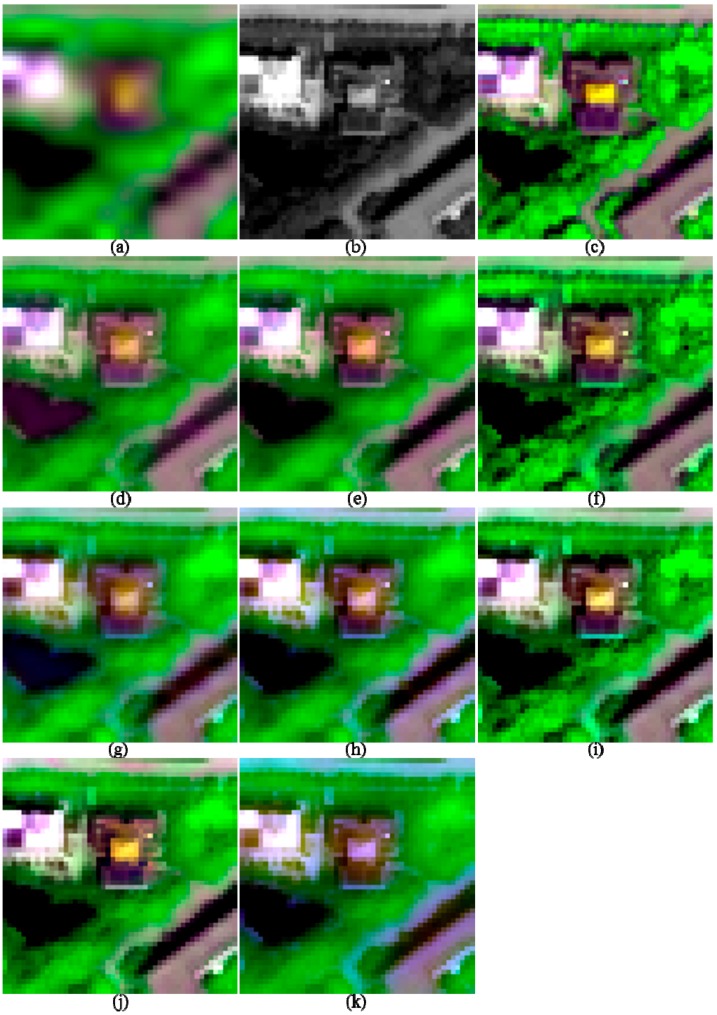
The original and 2-m fused images of I3, shown in band 5-7-2 composition. (**a**) 8-m MS; (**b**) 2-m PAN; (**c**) True 2-m MS; (**d**) GS; (**e**) GSA; (**f**) HR; (**g**) HCS; (**h**) ATWT; (**i**) GLP_ESDM; (**j**) GLP_ECBD; and (**k**) NSCT_M2.

**Figure 6:**
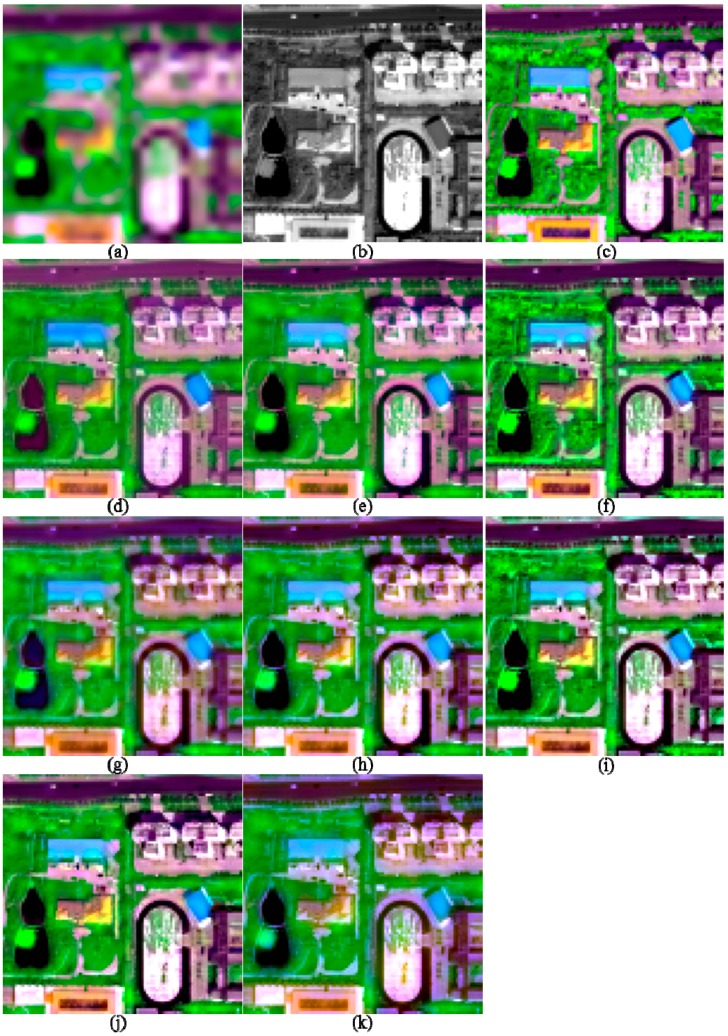
The original and 2-m fused images of I4, shown in band 5-7-2 composition. (**a**) 8-m MS; (**b**) 2-m PAN; (**c**) True 2-m MS; (**d**) GS; (**e**) GSA; (**f**) HR; (**g**) HCS; (**h**) ATWT; (**i**) GLP_ESDM; (**j**) GLP_ECBD; and (**k**) NSCT_M2.

The image quality indices of the fusion products of the two suburban images are shown in Table 3. Similarly, parts of the fusion products of the two images are shown in Figure 5 and Figure 6, respectively.

The GSA method offers best performances in terms of ERGAS, SAM, and Q8, whereas the GS method provides the highest SCC values, for the two suburban images. The NSCT_M2 method gives the poorest performances in terms of ERGAS, SAM, and Q8. Among the MRA methods, the GLP_ECBD method offers the highest Q8 value, whereas the ATWT method provides the highest SCC values. Although the HR method provides Q8 values slightly lower than the highest value provided by the GSA method, the former offers the lowest SCC value among the CS methods. The HR method also provides the highest SAM values among the CS methods. Although the HR method gives the best performances for the two urban images, it offers relative poor performances for the two suburban images. 

Visual comparisons of the fusion products of the two suburban images show that obvious spectral distortions can be found from the water-body and shadow covered regions in the fused images generated by GS, HCS, ATWT, and NSCT_M2 methods. In addition, the fusion products of the HCS and NSCT_M2 methods are significant more blurred than other products, due to few spatial details are injected into the fused images. The fusion products generated by HR, GLP_ESDM, and GLP_ECBD methods are more sharpened and yield lower spectral distortions than other fusion products. Although the GSA method gives the best performance in terms of Q8, the corresponding fusion products seem to be more blurred than the fusion products generated by the HR, GLP_ESDM, GLP_ECBD methods. Although the GS method offers the highest SCC values, the two GS-fused images show significant spectral distortions. The two HR-fused images also show very blurred boundaries between vegetation and non-vegetation objects, which may contribute to the relative low SCC values and high SAM values provided by this method. According to the quality indexes and visual inspection, the GLP_ECBD method gives better performances than other methods for the two suburban images.

#### 3.3.3. Assessment for the Two Rural Images

**Figure 7:**
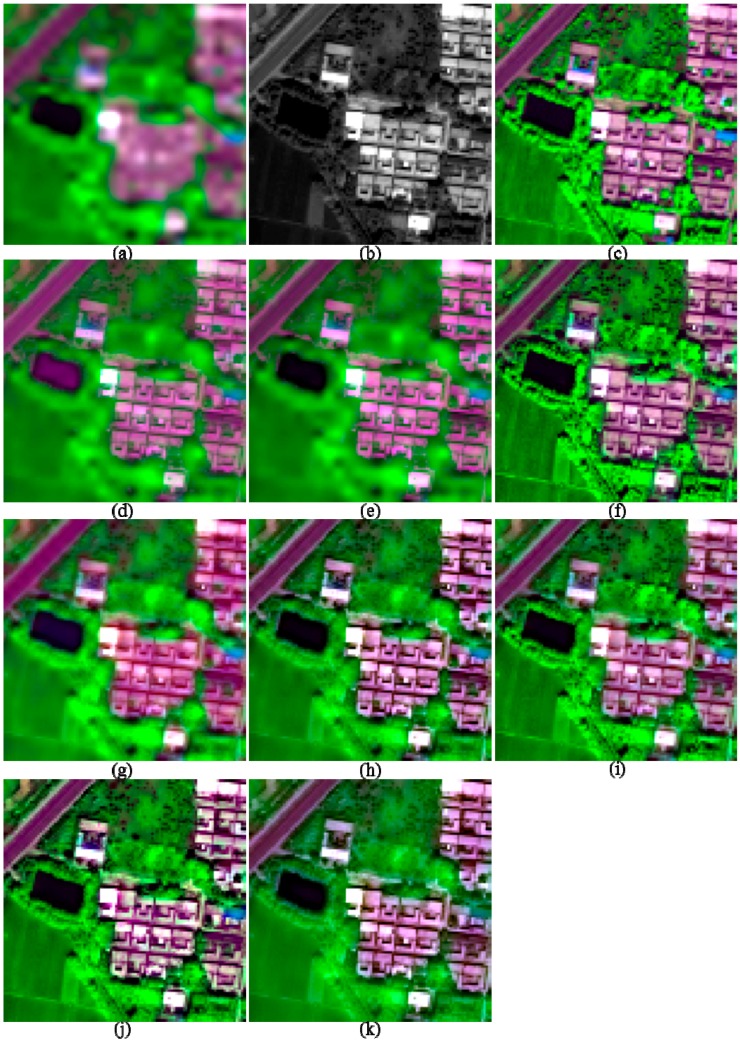
The original and 2-m fused images of I5, shown in band 5-7-2 composition. (**a**) 8-m MS; (**b**) 2-m PAN; (**c**) True 2-m MS; (**d**) GS; (**e**) GSA; (**f**) HR; (**g**) HCS; (**h**) ATWT; (**i**) GLP_ESDM; (**j**) GLP_ECBD; and (**k**) NSCT_M2.

**Figure 8:**
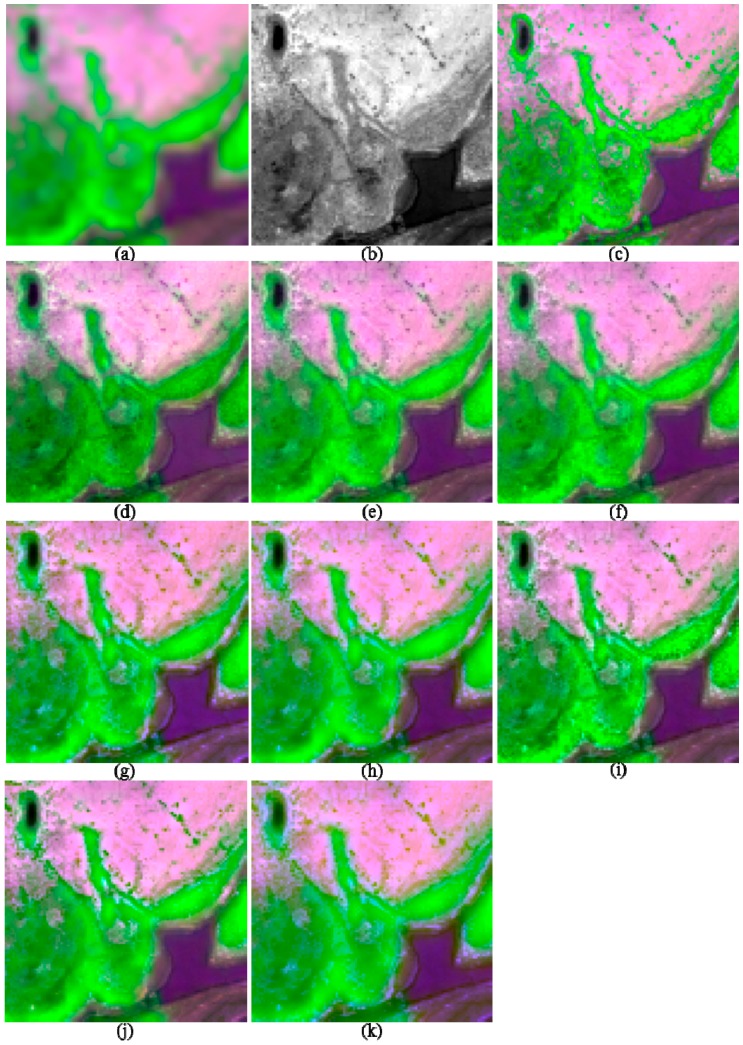
The original and 2-m fused images of I6, shown in band 5-7-2 composition. (**a**) 8-m MS; (**b**) 2-m PAN; (**c**) True 2-m MS; (**d**) GS; (**e**) GSA; (**f**) HR; (**g**) HCS; (**h**) ATWT; (**i**) GLP_ESDM; (**j**) GLP_ECBD; and (**k**) NSCT_M2.

The image quality indices of the fusion products of the two rural images are shown in Table 4, whereas the sub-images of fusion products of the two images are shown in Figure 7 and Figure 8, respectively.

The eight fusion methods give different performances for I5 and I6, which may due to the fact that the land cover types of the two scenes are obviously different. For I5, the HR and GLP_ESDM methods give the highest Q8 and SCC values; the GS and NSCT_M2 methods provide the lowest Q8 values; the GSA method yields the lowest SCC values. However, for I6, the HR, GLP_ECBD, and ATWT methods offer the highest Q8 values, whereas the ATWT, NSCT_M2, and GLP_ECBD methods provide the highest SCC values. The HCS and GS methods provide the lowest Q8 and SCC values, respectively, for I6. The GLP_ESDM gives a slightly better performance than the GLP_ECBD method for I5. In contrast, the former offers a poorer performance than the latter for I6. 

Obvious spectral distortions can be observed from the fused images generated by the GS, GSA, and HCS methods for I5, especially for the water-body covered regions and rooftop regions. The fusion products generated by the GS, GSA, and HCS methods for I5 are also more blurred than other fusion products. Conversely, the fused image generated by GLP_ECBD for I5 seems to be too sharpened, due to over injection of the spatial details. Consequently, the HR and GLP_ESDM methods give the best performances for I5, with respect to both the quality indexes and visual inspection.

The eight fusion products for I6 show no significant differences. Among the eight fusion products for I6, the product generated by the NSCT_M2 method is the most blurred; whereas pixels corresponding to vegetation in the product generated by the GLP_ESDM method seem to be too light colored. The HR-fused image shows slightly more details than other products, especially in vegetation covered regions. With respect to the quality indexes and visual inspection, the HR, GLP_ECBD, and ATWT methods yield the best performances for I6.

### 3.4. Information Preservation

For each of the fusion products, a CC between an information index derived from the fused image and the same index derived from the corresponding reference MS image was calculated. The CC values for MBI (*C*_MBI_), NDVI (*C*_NDVI_) and NDWI (*C*_NDWI_) for fusion products of the six test datasets are listed in Table 5.

#### 3.4.1. *C*_MBI_

The fused products generated from the two urban (I1 and I2) and tow suburban (I3 and I4) images were assessed in terms of *C*_MBI_. All the fusion products of the two urban images offer *C*_MBI_ values that are significant higher than those of the up-sampled MS images (EXPs), indicating that all the fusion products show obvious improvements in terms of *C*_MBI_. This is due to the fact that spatial details extracted from the PAN bands were injected to produce these images. Generally, the performances of the eight methods in terms of *C*_MBI_ are consistent with those in terms of Q8 and SCC. The HR, GSA, and GS methods offer the highest *C*_MBI_ values, whereas the NSCT_M2 and HCS methods offer the lowest *C*_MBI_ values, for both the urban and suburban test images. The CS methods give slightly better performances than the MRA methods in terms of *C*_MBI_, for the four test images. This is consistent with the performances of the eight methods in terms of quality indexes, for the four test images. This is also consistent with the conclusion of previous studies, which imply that the fusion products generated by the CS methods offer good visual and geometrical impression [31]. 

Among the MRA methods, the GLP_ESDM method offers the best performance for the two urban images, whereas the GLP_ECBD method performs the best for the two suburban images, in terms of *C*_MBI_. This is also consistent with the performances of the two methods in terms of quality indexes. According to the assessment results with respect to the quality indexes and visual inspection, the HR, GSA, and GLP_ESDM methods outperform other methods for the two urban images, whereas the GLP_ECBD method gives the best performances for the two suburban images. Actually, although the four method give different performances vary different image scenes, they outperform the other methods for all the four images, in terms of *C*_MBI_, as well as the quality indexes and visual inspection. Consequently, the HR, GSA, GLP_ESDM, and GLP_ECBD methods may be the best choice for producing fused urban/suburban WV-2 images used for image interpretation and buildings extraction. 

#### 3.4.2. *C*_NDVI_ and *C*_NDWI_

The fusion products of all the six images were assessed using *C*_NDVI_, whereas only the fusion products for the two suburban images and the two rural images were assessed using *C*_NDWI_. We discuss the two indexes together because both of the two indexes measure the differences between inter-band relationships of the fused image and those of the reference MS image. In addition, both of them are related to the NIR bands. 

It can be observed that some of the fusion products offer *C*_NDVI_ or *C*_NDWI_ values that are lower than those of provided by the corresponding up-sampled MS images (EXPs), and the highest *C*_NDVI_ values are just slightly higher than those of the EXPs. This indicates that the fusion products show limited improvements of in terms of *C*_NDVI_ and *C*_NDWI_, which may be caused by the spectral distortions of the fused NIR bands. 

The HCS method offers relative high *C*_NDVI_ and *C*_NDWI_ values for all the test images. This may due to the fact that the inter-band relationships of up-sampled LSR MS bands are preserved in the corresponding HCS-fused images. Since the HCS-fused images show significant spectral distortions in terms of both quality indexes and visual inspection, it is regarded as offering poor performance for specific applications. Hence, we do not discuss about the performance of this method henceforth in this section.

For the two urban images, the GLP_ESDM, HR, and GS methods offer the highest *C*_NDVI_ and *C*_NDWI_ values. With respect to the fact the GS-fused images show obvious spectral distortions and only the GLP_ESDM method offers higher *C*_NDVI_ and *C*_NDWI_ values than those of the corresponding EXPs, the GLP_ESDM method is a better choice for producing fusion products that will be used in applications related to urban vegetation and water-bodies.

For I3 and I4, the GS, ATWT, and GLP_ECBD methods provide the highest *C*_NDVI_ and *C*_NDWI_ values, whereas the HR and NSCT_M2 methods offer the lowest *C*_NDVI_ and *C*_NDWI_ values. With respect to the fact that the GS and ATWT methods give poor performances in terms of quality indexes and visual inspection, the GLP_ECBD method give the best performances for the two suburban images in terms of NDVI and NDWI information preservation, as well as quality indexes and visual inspection. 

For I5, the GLP_ESDM, ATWT, and HR methods offer higher *C*_NDVI_ and *C*_NDWI_ values than other methods. With respect to the fact that the ATWT method gives poor performances in terms of quality indexes and visual inspection and only the GLP_ESDM method offers higher *C*_NDVI_ and *C*_NDWI_ values than those of the corresponding EXPs, the GLP_ESDM method is the better choice for rural WV-2 images with similar image objects with I5, in terms of NDVI and NDWI information preservation, as well as quality indexes and visual inspection.

For I6, the GLP_ECBD, ATWT, and GLP_ESDM methods provide higher *C*_NDVI_ and *C*_NDWI_ values than the other methods. With respect to the fact that the HR, GLP_ECBD, and ATWT methods outperform the other methods in terms of quality indexes and visual inspection only the GLP_ECBD method offers higher *C*_NDVI_ and *C*_NDWI_ values than those of the corresponding EXPs, the GLP_ECBD method is the best choice for rural WV-2 images with similar image objects with I6, in terms of NDVI and NDWI information preservation, as well as quality indexes and visual inspection.

## 4. Discussion

Generally, the comparisons of different pansharpening methods are performed by assessing fusion products using spectral and spatial quality indexes, as well as visual inspection. However, a good performance in terms of quality indexes and visual inspection does not always result in a good choice for different application purposes. The NDVI, NDWI, and MBI index, which are widely used in applications related land cover classification, the extraction of vegetation area, buildings, and water bodies, were employed in this study to evaluate the performances of the selected pansharpening methods in terms of the information presentation ability. In this study, the performances of eight selected state-of-art pan-sharpening methods were assessed using information indices (NDVI, NDWI and MBI), along with current image quality indices (ERGAS, SAM, Q2^n^ and SCC) and visual inspection, with six datasets from two WV-2 scenes.

### 4.1. General Performances of the Selected Pansharpening Methods

Generally, the HR, GSA, GLP_ESDM, and GLP_ECBD methods give better performances than the other methods, whereas the NSCT and HCS methods offer the poorest performances, for most of the test images, in terms of quality indexes and visual inspection. The four methods also give slightly different performances for images including different image objects. For example, the HR, GSA, GLP_ESDM methods give the best performances for the two urban images, whereas the GLP_ECBD provides the best performances for the two rural images. However, the fusion products of the four methods offer good visual quality for most images. Consequently, the HR, GSA, GLP_ESDM, and GLP_ECBD methods are good choices if the fused WV-2 images will be used for image interpretation. 

The results of the assessments using the three information indices show that the rank of the selected eight fusion methods in terms of *C*_MBI_ is a little similar with those in terms of Q8 and SCC. This may indicate that the assessment using only the quality indexes and visual inspection is sufficient for selecting a best fusion method for producing fused urban WV-2 images used for image interpretation and applications related to urban buildings. The order of eight methods for in terms of *C*_NDVI_ is similar with that in terms of *C*_NDWI_. This is due to the fact that both *C*_NDVI_ and *C*_NDWI_ measure the differences between the inter-band relationships of a fused image and those of the corresponding reference MS image. In contrast, the orders of the eight methods in terms of *C*_NDVI_ and *C*_NDWI_ are significant different from those in terms of Q8 and SCC. This indicates that a fusion method offering the best performance for a certain image in terms of quality indexes and visual inspection does not always provide the highest *C*_NDVI_ and *C*_NDWI_ values. Generally, the GLP_ESDM method outperforms the other methods for I1, I2 and I5, whereas the GLP_ECBD method provides the best performances for I3, I4 and I6, in terms of *C*_NDVI_ and *C*_NDWI_, as well as quality indexes and visual inspection. This indicates that the GLP_ESDM is the best choice for images with similar objects with I1, I2 and I5, whereas the GLP_ECBD is the best choice for images with similar objects with I3, I4 and I6, for producing fusion products used for applications related to vegetation or water-bodies. In addition, the fusion products show limited improvements in terms of *C*_NDVI_ and *C*_NDWI_. This indicates that it is hard for the fusion products to preserve the NDVI and NDWI information obtained from the corresponding up-sampled MS images. Consequently, it is necessary to evaluate fusion products using information indices (i.e., NDVI and NDWI) if fused WV-2 images will be used for applications related to vegetation and water-bodies. 

### 4.2. Effects for Different Spectral Ranges between the PAN and MS Bands

A noticeable point for the WV-2 is that the spectral range of the PAN band covers limited portion of the spectral ranges of the C, NIR1 and NIR2 bands. This results in relative low correlation coefficients between these bands and the PAN band. It is interesting to see the performances of the selected pansharpening methods on the two NIR bands and the C band of WV-2. In order to assess the spectral distortion of each fused band, the CC value between each fused band and the corresponding reference band was calculated for each fusion product. The CC values for the fusion products of I1, I3, I5 and I6 are shown in Table 6. The CC values of I2 and I4 are not presented because they are similar with those of I1 and I3, respectively. 

It can be seen from Table 6 that the CC values of the two NIR bands are significantly lower than those of the other bands for all the fusion products, indicating that the fused NIR bands show more spectral distortions than the other bands. This is caused by the relative low correlation coefficients between the two NIR bands and the PAN band. This is also revealed by previous studies, the higher the correlation between the PAN band and each MS band, the better the success of fusion [31]. Generally, the four CS methods offer higher CC values for the two NIR bands than the four MRA methods for most of the test images. This is consistent with the result of visual inspection of these fusion products. However, the two GLP-based methods, which provide good performances in terms of NDVI and NDWI information preservation, offer relative low CC values for the fused NIR1 band, due to the low CC between the PAN and the NIR1 band. This proves again that it is necessary to evaluate fusion products using information indices (i.e., NDVI and NDWI) if fused WV-2 images will be used for applications related to vegetation and water-bodies. 

### 4.3. How to Extend the Selected Pansharpening Methods to Other HSR Satellite Images

As introduced in the previous sections, the HR, GSA, GLP_ESDM, and GLP_ECBD methods are good choices for producing fused WV-2 images used for image interpretation and applications related to urban buildings. The two GLP-based methods outperform other methods for generating fused WV-2 images used for applications related to vegetation and water-bodies. It is interesting for the readers that whether these methods give similar performances to the sensors having a similar PAN spectral range with WV-2, such as GeoEye-1, and WorldView-3/4. 

Actually, the selected pansharpening methods can be categorized into two groups, according to the approaches employed to generate the synthetic PAN band $P_{L}$, which mainly contains the low-frequency component of the original PAN band. For the first group, $P_{L}$ is generated by applying filters to the original PAN band, or by up-sampling the degraded version of the original PAN band. In contrast, for the second group, the intensity image $I_{L}$, which can be seen as another approach for generating the synthetic PAN band $P_{L}$, is generated using the weighted combination of the LSR MS bands. The methods belong to the first group include HR, ATWT, NSCT and the two GLP-based methods, whereas the methods belong to the second group include GS, GSA and HCS methods. For the first group, the low-frequency component of $P_{L}$ has relative low correlations with the C, NIR1 and NIR2 bands, but has relative high correlations with the other spectral bands. This result in the fact that the details of the PAN band have relative high correlations with the B, G, Y, R and RE bands, but relative low correlations with the C, NIR1 and NIR2 bands. This may result in the fact that a large amount of spatial details are injected into the B, G, Y, R and RE bands, but only a small amount of the spatial details are injected into the C, NIR1 and NIR2 bands, especial for the case the injection gains are determined considering the relationship between each MS band and the PAN band. For the second group, the low-frequency component of the intensity image is related or partly related to the C, NIR1 and NIR2 bands. This may result in the fact that the low-frequency component of the C, NIR1 and NIR2 bands may be injected into the B, G, Y, R and RE bands, and hence may lead to spectral distortions of these bands. An exception occurs for GSA, since the intensity image $I_{L}$ employed the GSA method have low CC with the C, NIR1 and NIR2 bands, due to the weights *w_i_* obtained using Equation (5) are very low for these bands. 

According to the introduction about the algorithms of the selected methods, different injection gains $g_{i}$ are employed by these methods. The GS and GSA methods use a band-dependent model considering the relationship between each MS band and the PAN band. The GSA method outperform the GS method due to the intensity image $I_{L}$ employed the former have low CC values with the C, NIR1 and NIR2 bands. It can be seen from Table 6 that the CC values for the B, G, Y, R, and RE bands of the GSA-fused images are significantly higher than those of the GS-fused image. The HR method uses the SDM model, which is also band-dependent. The ATWT method employs a simple additive injection model with weights for each band equal to 1, whereas the two GLP-based methods use the ESDM and ECBD models, respectively. Among these models, only the ESDM and ECBD models consider the local dissimilarity between the MS and PAN bands. According to the experimental results, the two GLP-based methods give good performances in terms of NDVI and NDWI information preservation. This may due to the fact that only the ESDM and ECBD models consider the local dissimilarity between the MS and PAN bands. It is also demonstrated by previous studies that local dissimilarity between the MS and PAN bands should be considered by pansharpening methods to reduce spectral distortions.

As a result of the above analyses about the algorithms of the selected pansharpening methods, we can obtain the following conclusions. Firstly, for the spectral bands with relative high correlations with the PAN band, the synthetized PAN band should be obtained using the original PAN band and the injection gains should considering the relationship between each MS band and the PAN band. Secondly, for the spectral bands with relative low correlations with the PAN band, further experiments should be designed to evaluate which approach is better for generating the synthetized PAN band. However, there is no doubting that local dissimilarity between the MS and PAN bands should be considered for the fusion of these bands, i.e., the NIR band, especially for the case that the fused images will be used in applications related to vegetation and water-bodies. 

According to the analysis, we can conclude that the GSA, HR, GLP_ESDM, and GLP_ECBD methods can also provide good performances for similar sensors, such as GeoEye-1, WorldView-3, WorldView-4, for the cases that the fusion products will be used in image interpretation or urban buildings. Actually, it is proved by previous studies that the performances of these newly proposed methods are sensor independent [30]. However, for the case that the fusion products will be used in applications related to vegetation or water-bodies, the GLP-ESDM and GLP_ECBD methods or other fusion methods consider local dissimilarity between the MS and PAN bands are better choices.

## 5. Conclusions

The performances of eight state-of-art pan-sharpening methods for WV-2 imagery were assessed using information indices (NDVI, NDWI, and MBI), along with current image quality indices (ERGAS, SAM, Q2^n^, and SCC) and visual inspection, with six WV-2 datasets. The main findings and conclusions derived from our analyses are as follows:

(1) Generally, the HR, GSA, GLP_ESDM and GLP_ECBD methods give better performances than the other methods, whereas the NSCT and HCS methods offer the poorest performances, for most of the test images, in terms of quality indexes and visual inspection. Some of the fusion products generated by the GS and ATWT methods show significant spectral distortions. In addition, the performances of the eight methods in terms of *C*_MBI_ are consistent with those in terms of Q8 and SCC. Consequently, the HR, GSA, GLP_ESDM, and GLP_ECBD methods are good choices if the fused WV-2 images will be used for image interpretation and applications related to urban buildings. The four methods can also provide good performances for other WV-2 image scenes, for producing fused images used for image interpretation.
(2) The order of the pansharpening methods in terms of *C*_NDVI_ is consistent with that in terms of *C*_NDWI_. This is because both of the two indexes measure the differences between inter-band relationships of the fused image and those of the reference MS image, and both of them are related to the quality of the fused NIR1 bands. The GLP_ESDM method offers higher *C*_NDVI_ and *C*_NDWI_ values for I1, I2 and I5, whereas the GLP_ECBD method provides higher *C*_NDVI_ and *C*_NDWI_ values for I3, I4 and I6, as well as good performances in terms of quality indexes and visual inspection. Consequently, the GLP_ESDM and GLP_ECBD methods are better than other methods, if the fused WV-2 images will be used for applications related to vegetation and water-bodies. However, for this case, it is better to select a best method by comparing the indexes *C*_NDVI_ and *C*_NDWI_, as well as quality indexes and visual inspection, since the GLP_ESDM and GLP_ECBD methods may give different performances for images with different land cover objects.
(3) According to the experimental results of this work and the analyses the algorithms of the selected pansharpening methods, we can offer two suggestions for the fusion of images obtained by sensors similar with WV-2, such as Geoeye-1 and Worldview-3/4. Firstly, for the spectral bands with relative high correlations with the PAN band, the synthetized PAN band should be obtained using the original PAN band and the injection gains should considering the relationship between each MS band and the PAN band. The HR, GSA, GLP_ESDM, and GLP_ECBD method also can offer good performances for scenes obtained by GeoEye-1 and Worldview-3/4, for producing fused images used for interpretation and applications related to urban buildings. Secondly, for the spectral bands with relative low correlations with the PAN band, local dissimilarity between the MS and PAN bands should be considered for the fusion of these bands, i.e., the NIR band, especially for the case that the fused images will be used in applications related to vegetation and water-bodies.

## Figures and Tables

**Table 1:** The description of the selected six datasets.

Image	Location	Type	Objects	Information Indices
I1	Beijing	Urban	High buildings, squares, roads, vegetation, shadows	MBI, NDVI
I2	Beijing	Urban	Moderate buildings, squares, roads, vegetation, shadows	MBI, NDVI
I3	Pingdingshan	Suburban	Low buildings, squares, roads, vegetation, shadows, water bodies	MBI, NDVI, NDWI
I4	Pingdingshan	Suburban	Low buildings, squares, roads, vegetation, shadows, water bodies	MBI, NDVI, NDWI
I5	Pingdingshan	Rural	Building, roads, farms, water bodies	NDVI, NDWI
I6	Pingdingshan	Rural	Vegetation, water bodies, bare soils	NDVI, NDWI

**Table 2:** Quality indices of the fused images generated by the selected algorithms for the two urban images.

Image	Method	ERGAS	SAM	Q8	SCC
I1	GS	1.64	2.19	0.957	0.911
	GSA	1.28	1.92	0.974	0.908
	HR	1.26	1.76	0.977	0.911
	HCS	1.95	2.58	0.897	0.881
	ATWT	2.09	2.72	0.879	0.863
	GLP_ESDM	1.56	2.05	0.956	0.898
	GLP_ECBD	1.93	2.36	0.956	0.888
	NSCT_M2	2.22	3.66	0.849	0.861
	EXP	1.64	2.19	0.857	0.582
I2	GS	2.70	3.79	0.909	0.844
	GSA	2.22	3.45	0.946	0.839
	HR	2.20	3.14	0.951	0.849
	HCS	2.87	3.99	0.850	0.809
	ATWT	2.76	4.02	0.861	0.799
	GLP_ESDM	2.51	3.47	0.916	0.824
	GLP_ECBD	3.14	4.26	0.905	0.785
	NSCT_M2	3.04	5.03	0.831	0.795
	EXP	3.84	3.99	0.797	0.499

**Table 3:** Quality indices of the fused images generated by the selected algorithms for the two suburban images.

Image	Method	ERGAS	SAM	Q8	SCC
I3	GS	1.31	1.90	0.908	0.885
	GSA	1.03	1.76	0.942	0.883
	HR	1.38	2.23	0.927	0.849
	HCS	1.28	1.98	0.881	0.871
	ATWT	1.21	1.93	0.907	0.872
	GLP_ESDM	1.39	1.91	0.902	0.835
	GLP_ECBD	1.33	1.91	0.921	0.856
	NSCT_M2	1.39	2.41	0.887	0.869
	EXP	1.62	1.98	0.837	0.796
I4	GS	1.74	2.85	0.871	0.841
	GSA	1.43	2.74	0.919	0.831
	HR	1.84	3.25	0.893	0.779
	HCS	1.74	3.01	0.850	0.817
	ATWT	1.66	2.98	0.868	0.821
	GLP_ESDM	1.82	2.82	0.876	0.796
	GLP_ECBD	1.79	2.88	0.892	0.805
	NSCT_M2	1.88	3.64	0.843	0.818
	EXP	2.21	3.01	0.759	0.669

**Table 4:** Quality indices of the fused images generated by the selected algorithms for the two rural images.

Image	Method	ERGAS	SAM	Q8	SCC
I5	GS	1.66	2.41	0.822	0.838
	GSA	1.56	2.66	0.887	0.799
	HR	1.32	2.06	0.914	0.882
	HCS	1.61	2.23	0.837	0.861
	ATWT	1.59	2.37	0.853	0.857
	GLP_ESDM	1.49	1.99	0.875	0.874
	GLP_ECBD	1.90	2.45	0.874	0.845
	NSCT-M2	1.71	2.83	0.829	0.855
	EXP	2.30	2.23	0.748	0.665
I6	GS	1.88	2.95	0.762	0.756
	GSA	1.16	1.75	0.857	0.785
	HR	1.08	1.60	0.873	0.812
	HCS	1.05	1.51	0.735	0.848
	ATWT	0.97	1.49	0.858	0.868
	GLP_ESDM	1.12	1.52	0.857	0.809
	GLP_ECBD	0.99	1.44	0.868	0.854
	NSCT-M2	1.10	1.77	0.820	0.865
	EXP	1.09	1.51	0.818	0.849

**Table 5:** The CC values for the information indices for the fused images generated by the selected eight algorithms.

Method	*C*_MBI_				*C*_NDVI_						*C*_NDWI_			
	I1	I2	I3	I4	I1	I2	I3	I4	I5	I6	I3	I4	I5	I6
GS	0.973	0.972	0.969	0.934	0.922	0.877	0.929	0.891	0.912	0.946	0.915	0.858	0.876	0.950
GSA	0.975	0.979	0.981	0.959	0.915	0.855	0.924	0.879	0.884	0.966	0.908	0.853	0.830	0.977
HR	0.976	0.978	0.977	0.950	0.923	0.885	0.899	0.816	0.927	0.969	0.876	0.780	0.895	0.981
HCS	0.962	0.949	0.923	0.848	0.926	0.883	0.917	0.880	0.940	0.974	0.911	0.859	0.917	0.986
ATWT	0.965	0.956	0.934	0.882	0.911	0.849	0.928	0.890	0.928	0.975	0.912	0.851	0.915	0.986
GLP_ESDM	0.965	0.960	0.945	0.899	0.928	0.887	0.915	0.871	0.942	0.972	0.901	0.841	0.922	0.984
GLP_ECBD	0.964	0.957	0.961	0.926	0.892	0.811	0.925	0.876	0.906	0.977	0.909	0.851	0.860	0.988
NSCT_M2	0.965	0.948	0.905	0.842	0.860	0.778	0.898	0.862	0.896	0.968	0.873	0.800	0.871	0.982
EXP	0.825	0.798	0.829	0.783	0.926	0.883	0.917	0.880	0.940	0.974	0.911	0.859	0.917	0.986

**Table 6:** The CC values for the fused bands for I1, I3, I5 and I6.

Image	Method	CC								
		C	B	G	Y	R	RE	NIR1	NRI2	Avg
**I1**	GS	0.985	0.985	0.988	0.992	0.990	0.990	0.978	0.978	0.986
	GSA	0.987	0.987	0.991	0.995	0.993	0.992	0.978	0.977	0.988
	HR	0.990	0.990	0.993	0.996	0.994	0.993	0.977	0.977	0.989
	HCS	0.819	0.945	0.982	0.989	0.987	0.984	0.970	0.969	0.956
	ATWT	0.849	0.931	0.985	0.991	0.986	0.987	0.974	0.971	0.959
	GLP_ESDM	0.959	0.979	0.986	0.990	0.990	0.988	0.975	0.973	0.980
	GLP_ECBD	0.982	0.982	0.986	0.989	0.986	0.985	0.967	0.959	0.980
	NSCT_M2	0.805	0.906	0.985	0.993	0.987	0.988	0.971	0.965	0.950
	EXP	0.952	0.948	0.944	0.942	0.939	0.928	0.917	0.916	0.936
**I3**	GS	0.958	0.957	0.962	0.967	0.962	0.952	0.899	0.903	0.945
	GSA	0.977	0.977	0.986	0.990	0.984	0.977	0.906	0.906	0.963
	HR	0.976	0.975	0.983	0.986	0.980	0.969	0.830	0.823	0.940
	HCS	0.836	0.946	0.977	0.979	0.969	0.968	0.892	0.893	0.933
	ATWT	0.918	0.932	0.980	0.982	0.975	0.971	0.900	0.898	0.945
	GLP_ESDM	0.919	0.964	0.978	0.985	0.980	0.966	0.842	0.844	0.935
	GLP_ECBD	0.962	0.963	0.975	0.982	0.975	0.965	0.876	0.861	0.945
	NSCT_M2	0.886	0.906	0.980	0.978	0.969	0.968	0.872	0.863	0.928
	EXP	0.922	0.921	0.921	0.923	0.920	0.910	0.886	0.885	0.911
**I5**	GS	0.958	0.951	0.945	0.947	0.953	0.908	0.868	0.875	0.926
	GSA	0.969	0.964	0.963	0.962	0.967	0.981	0.949	0.957	0.964
	HR	0.968	0.966	0.974	0.968	0.967	0.984	0.954	0.965	0.968
	HCS	0.739	0.892	0.954	0.966	0.967	0.984	0.962	0.973	0.930
	ATWT	0.901	0.924	0.976	0.967	0.965	0.987	0.966	0.975	0.958
	GLP_ESDM	0.913	0.962	0.975	0.970	0.970	0.984	0.949	0.963	0.961
	GLP_ECBD	0.942	0.935	0.959	0.967	0.968	0.985	0.964	0.974	0.962
	NSCT_M2	0.858	0.888	0.970	0.960	0.957	0.981	0.957	0.968	0.943
	EXP	0.940	0.932	0.923	0.925	0.931	0.976	0.966	0.974	0.946
**I6**	GS	0.957	0.959	0.967	0.967	0.966	0.948	0.802	0.800	0.921
	GSA	0.970	0.974	0.985	0.982	0.980	0.963	0.750	0.747	0.919
	HR	0.979	0.980	0.987	0.987	0.985	0.969	0.858	0.854	0.950
	HCS	0.879	0.966	0.978	0.971	0.964	0.936	0.848	0.846	0.924
	ATWT	0.896	0.924	0.980	0.979	0.971	0.950	0.858	0.862	0.928
	GLP_ESDM	0.905	0.962	0.976	0.978	0.979	0.955	0.869	0.848	0.934
	GLP_ECBD	0.956	0.960	0.972	0.971	0.967	0.945	0.790	0.829	0.924
	NSCT_M2	0.873	0.905	0.982	0.978	0.968	0.953	0.801	0.829	0.911
	EXP	0.904	0.899	0.893	0.899	0.900	0.788	0.747	0.748	0.848
